# A digital cognitive behavioral therapy program culturally adapted for Spanish-speaking individuals with alcohol use disorder: a stage 1 randomized clinical trial

**DOI:** 10.3389/fdgth.2026.1729049

**Published:** 2026-04-21

**Authors:** Brian D. Kiluk, Manuel Paris, Bryan Benitez, Yudilyn Jaramillo, Oscar Rojas-Perez, Luis Anez, Tami L. Frankforter, Charla Nich, Michelle Silva

**Affiliations:** Department of Psychiatry, Yale School of Medicine, New Haven, CT, United States

**Keywords:** alcohol use disorder (AUD), cognitive behavioral therapy (CBT), culturally adapted CBT, digital CBT, Spanish speakers

## Abstract

**Background:**

Digital formats are an important tool for making evidence-based therapies for alcohol use, such as cognitive behavioral therapy (CBT), more broadly available, yet only a small percentage are available in Spanish and none with evidence from effectiveness studies with Spanish-speaking individuals. This study evaluated the feasibility and efficacy of adding a culturally-adapted, web-based CBT program for Spanish-speakers, known as *CBT4CBT-SA*, to community-based outpatient care for alcohol use disorder.

**Methods:**

We conducted a 2-arm, unblinded, parallel-group randomized clinical trial among 51 primary Spanish-speaking adults seeking outpatient treatment for alcohol use. Participants were randomly assigned to either standard treatment (ST), or ST plus access to the CBT4CBT-SA program. Frequency of alcohol use was measured during an 8-week treatment period, and at monthly intervals at 1-, 3-, and 6-month follow-up. The primary outcome was the percentage of days abstinent (PDA) from alcohol by week during the 8-week period. Other outcomes included change in consequences from alcohol, coping strategies, and knowledge of CBT concepts.

**Results:**

Rates of treatment completion (63%) and data availability (84% of randomized sample at 6-month follow-up) were high. Results of random effects regression analyses did not indicate a significant change in PDA in the full sample. Among the subsample of treatment completers, those assigned to ST + CBT4CBT-SA had a greater increase in PDA compared to ST during the 8-week treatment (treatment by week, *t*_258_ = 2.56, *p* = .01), as well as across the full 8-month study period (treatment by month, *t*_239.85_ = 2.50, *p* = .01). While there was a subsequent reduction in negative consequences from alcohol during the treatment period, it did not differ by treatment condition.

**Conclusion:**

This culturally adapted, web-based version of CBT targeting alcohol use for Spanish speakers appeared acceptable with preliminary efficacy at improving alcohol use outcomes when added to standard outpatient treatment. Future studies are warranted to evaluate efficacy in a larger sample and with differing levels of clinical support.

## 1 Introduction

The population of individuals identifying as Hispanic/Latino currently represent 19% of the total U.S. population, making it the nation's second largest racial or ethnic group after non-Hispanic Whites (1) (NB: we use the term Hispanic hereafter to include individuals of Mexican, Puerto Rican, Cuban, Dominican, Central and South American, and Spanish descent, regardless of race). Although recognized as the country's largest ethnic minority group, there is ample evidence that Hispanic individuals experience circumstances that limit their access to culturally and linguistically appropriate evidence-based behavioral health treatments, compromising treatment outcomes (2–5). This is highly evident in our limited ability to adequately recognize and treat alcohol use disorder (AUD) among Hispanic individuals (6, 7). Compared to non-Hispanic White adults, Hispanic individuals are more likely to engage in heavier drinking, suffer from alcohol-associated liver disease, and experience more adverse social and legal consequences (8–11). Hispanic adults in need of alcohol treatment are less likely to use specialty alcohol treatment services compared to White adults (6), and when they do enter treatment, they are among the highest risk for dropout (12).

Limited English proficiency is a frequently cited challenge faced by the Hispanic patient population, associated with dissatisfaction with the healthcare system and lower use of mental health care (13, 14). Spanish-speaking Hispanics are considerably less likely to receive treatment compared to primarily English-speaking Hispanics, who have access patterns similar to non-Hispanic White individuals (15). There is evidence to suggest that treatment programs' ability to incorporate linguistic and cultural norms of Hispanics in treatment can improve access, duration, and treatment completion rates (16, 17). Also, studies have found increased treatment effectiveness when culturally appropriate interventions delivered in Spanish are integrated with elements of cognitive behavioral therapy (CBT) (18, 19). Yet many programs are unlikely to have the necessary staffing and resources to offer linguistic and culturally-adapted evidence-based treatments such as CBT, requiring individuals to travel longer distances to access Spanish-language services (20).

Digital interventions (those delivered through web-based, mobile, or other software platforms) have emerged as a promising strategy to address barriers to care among racial and ethnic minority communities (21). Benefits of using a digital platform for intervention delivery include improving access for those with geographic and logistical barriers, those who do not seek treatment due to low readiness, discrimination or stigma, or those who may experience poverty and environmental violence that may impede access to in-person treatment (22, 23). Also, they offer significant advantages of standardization and consistent quality, reduction of cost and clinician time, and potential 24/7 availability (24). Given the growing acceptance of digital health technologies to extend healthcare (25), and the increasing rates of internet use among Hispanics and those who are Spanish dominant (26), the conversion of linguistic and culturally-adapted interventions to digital platforms may offer a solution to the challenge of access to evidence-based treatments for AUD among Spanish-speaking Hispanics.

CBT is an evidence-based treatment for a range of mental health conditions, including AUD (27), and is one of the most common approaches provided through digital interventions (28, 29). We developed a computer-based version of CBT for substance use disorders, called CBT4CBT (*Computer-Based Training for Cognitive Behavioral Therapy*) that has demonstrated efficacy at improving substance use outcomes when delivered as an adjunct to standard treatment in outpatient treatment settings (30–34). This includes efficacy of a culturally-adapted version to address drug use among monolingual Spanish-speaking Hispanics [CBT4CBT-S; (35)]. Described in detail elsewhere (36), the process of adapting CBT4CBT for Spanish-speakers involved modifications at the program and content level that included changes in teaching style, level of interaction, and format to make the educational subject matter more appealing and relevant to monolingual Spanish-speakers seeking treatment for substance use and mental health disorders. While the CBT4CBT-S program was designed to address any type of drug use, including alcohol, most of the content (e.g., animations, video-based character vignettes) was targeted toward illicit drug use. Although there are commonalities across substance use disorders, there are also differences between those with AUD compared to other substance use disorders in terms of patterns of use, social characteristics, and treatment goals (37, 38) that may influence treatment response. As such, we developed a new CBT4CBT-S version targeting alcohol use (CBT4CBT-Spanish Alcohol; CBT4CBT-SA) to address the need for interventions specific for AUD, which parallels our separate versions of CBT4CBT in English targeting drug (33) or alcohol use (34). In addition, research has shown a link between exposure to traumatic events, the development of post-traumatic stress disorder (PTSD), and alcohol use (39). Given that Hispanic adults have been found to experience unique psychosocial circumstances that heighten their risk of traumatic exposures, the CBT4CBT-SA version integrated content on the topic of trauma and its effects on alcohol use and mental health symptoms (40).

While digital interventions offer tremendous promise in terms of expanding access to evidence-based treatment for underserved populations, they must be sufficiently validated in order to achieve the benefits (41). The evidence standards for establishing efficacy of these tools should be the same as those for behavioral or pharmacologic treatments for mental health, such as through high-quality RCTs. Yet the current landscape of digital mental health technologies is marked by a striking lack of evidence supporting their efficacy (42), with only a small percentage of commercially available apps in Spanish and none with evidence from effectiveness studies in Spanish-speaking individuals (43). The purpose of the current study was to evaluate the feasibility and efficacy of adding the newly created CBT4CBT-SA targeting alcohol use to community-based outpatient care for AUD in a Stage 1 RCT. Given prior evidence supporting other versions of CBT4CBT, including CBT4CBT-S, as an adjunct to outpatient standard care for addiction, we hypothesized that CBT4CBT-SA plus standard care would be significantly more effective than standard care alone at increasing alcohol abstinence.

## 2 Methods

### 2.1 Overview

This was a 2-arm, unblinded, parallel-group RCT conducted between August 2019 and August 2023. The trial was approved by the Yale University Institutional Review Board and preregistered at clinicaltrials.gov—NCT03474588. Primary Spanish-speaking individuals seeking outpatient treatment for alcohol use were randomly assigned to receive either standard treatment (ST) at the outpatient facility (i.e., treatment as usual), or ST plus access to the CBT4CBT-SA program. Frequency of alcohol use was measured weekly during an 8-week treatment period, and at monthly intervals at 1-, 3-, and 6-month follow-up interviews. The primary outcome was the percentage of days abstinent (PDA) from alcohol by week during the 8-week period with durability of effects evaluated during the 6-month follow-up. The trial was powered to detect a medium effect (Cohen's *d* = 0.35) on primary outcome during the active treatment phase, which required a sample size of 102. All research and treatment visits were conducted remotely from March 2020 to July 2021 due to COVID-19 pandemic restrictions on in-person visits.

### 2.2 Participants

We recruited participants from community-based outpatient treatment facilities providing services in Spanish in the greater New Haven area. Participants included those who: (1) were at least 18 years old; (2) met current DSM-5 criteria for AUD with self-reported alcohol use in past month; (3) spoke Spanish as their principal or preferred language; (4) were deemed psychiatrically and medically stable for 8-weeks of outpatient treatment; and (5) were willing to be randomized to a treatment condition and provide locator information for follow-up. Individuals were excluded who: (1) had an untreated bipolar or schizophrenic disorder; (2) had a current legal case pending such that incarceration was imminent during the 8-week period; or (3) met DSM-5 criteria for another current substance use disorder (other than nicotine).

**Figure 1:**
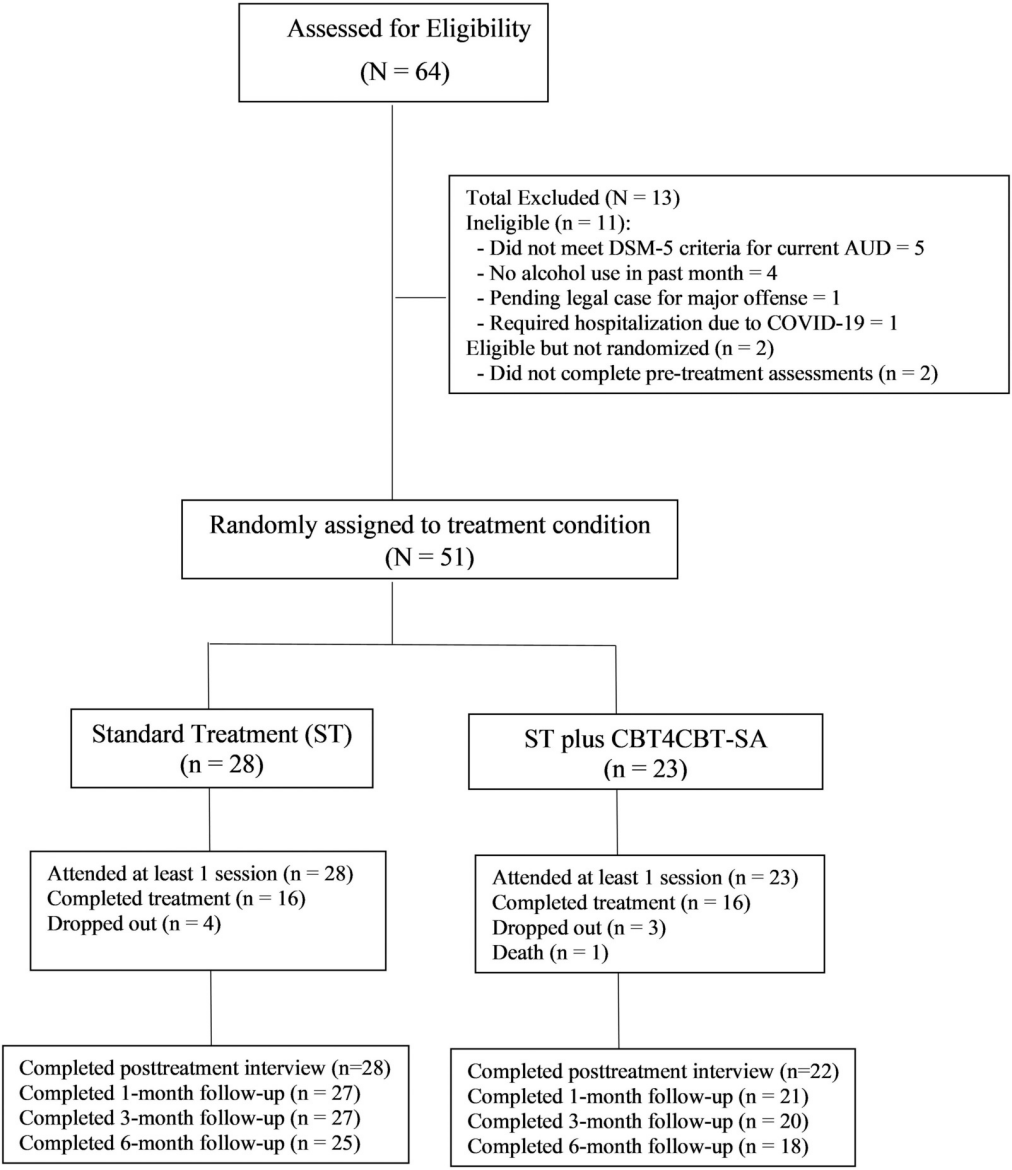
CONSORT diagram of participant flow.

See Figure 1 for a CONSORT diagram illustrating the flow of participants through the trial. A total of 64 individuals expressed interest, provided written informed consent, and were screened for eligibility. Of these, 51 were eligible, completed baseline assessments, and were randomly assigned to a treatment condition using a computerized urn randomization program to balance groups with respect to sex, education (completed high school; yes/no), severity of alcohol use (based on AUDIT score), level of familiarity with computers, and whether they met criteria for PTSD (yes/no).

### 2.3 Treatments

#### 2.3.1 Standard treatment as usual (ST)

Participants assigned to this condition received standard treatment at one of the community-based outpatient facilities providing behavioral health services for Spanish speakers. This typically consisted of weekly individual and/or group counseling in Spanish provided by masters-level or certified counselors. Counselors were permitted to use their usual counseling approach (e.g., motivational, skills-based, harm reduction) in group or individual sessions for participants in this study. All participants were provided with access to ancillary services through the treatment facility, which typically included psychiatric, pharmacologic, case management, and emergency services.

#### 2.3.2 ST plus CBT4CBT-SA

In addition to standard treatment, participants assigned to this condition were provided with access to the CBT4CBT-SA program, which could be accessed on-site using a dedicated laptop/tablet or off-site with their own device (cellular data-enabled tablets were provided for participants during the COVID-19 pandemic for those without a device). The program is self-guided; counselors providing ST in this condition were not involved with the delivery of CBT4CBT-SA. Bilingual and bicultural research staff provided the participant with unique credentials to login to the web-based program and guided them through the initial login process to assure they knew how to use the program and address any questions. Participants were asked to complete all 7 modules sequentially during the 8-week treatment period, with the option to repeat material as often as desired.

Described in more detail elsewhere (32, 44, 45), the original CBT4CBT program was designed as a highly engaging version of CBT to convey key skills via a range of media (e.g., video, graphics, audio instruction, interactive exercises). It uses video-based examples to emphasize learning of targeted behavioral, cognitive, and affective strategies, with emphasis on modeling skill usage from examples of individuals (professional actors) in a range of realistic situations. Interactive exercises reinforce patients' understanding of targeted skills, as well as brief comprehension quizzes and practice assignments to encourage implementation and enhance durability of effects. The program includes 7 core lessons (i.e., modules) each covering a core cognitive and behavioral strategy for addressing alcohol use, as based on CBT therapist manuals (46, 47). The CBT4CBT-S program included adaptations at the program and content level to incorporate Hispanic cultural values and concepts (e.g., respect—*respeto;* trust—*confianza;* gender-specific values—*machismo;* family orientation—*familismo;* interpersonal relationships—*personalismo)*, while maintaining consistency with CBT principles (36). Using an entertainment-education method, a *telenovela* format provided the platform for teaching cognitive and behavioral coping strategies in an engaging manner with cultural values integrated throughout the storyline and characters. For the CBT4CBT-SA version, new characters and alcohol-focused scenarios were created to convey coping strategies to address alcohol use, as well as additional content on the topic of trauma and alcohol use. See Supplementary Materials for screenshots of the CBT4CBT-SA program.

The program has an underlying data structure that tracks, for each user account, the number of modules completed, the amount of time (in minutes) spent accessing the program, responses to quiz questions, and completion of practice exercises.

### 2.4 Assessments

A bilingual and bicultural research assistant administered assessments in-person or remotely at baseline, weekly during the treatment period, at the end of the 8-week treatment, and at 1-, 3-, and 6-months following the end of treatment period. Assessments included those with Spanish translations already available or those validated in previous work with Spanish-speaking populations (48, 49).

#### 2.4.1 Structured clinical interview for DSM-5 (SCID-5)

The SCID-5 is an interview-based assessment administered at screening to determine eligibility with respect to alcohol and psychiatric diagnoses. The substance use section was repeated at the end-of-treatment (week 8) and 6-month follow-up to assess endorsement of DSM-5 criteria over the past 30 days (50).

#### 2.4.2 Alcohol use disorders identification test (AUDIT)

The AUDIT is a self-report assessment used to measure hazardous drinking and negative consequences over the past year, with total scores ranging from 0 to 40; scores greater than 14 indicate increased likelihood of moderate to severe AUD. Internal consistency of the Spanish-version of the AUDIT in this sample was good (Cronbach's *α* = 0.82) (51, 52).

#### 2.4.3 Substance use calendar

The Substance Use Calendar is a calendar-based Timeline Follow Back method (53, 54) that measured self-reported frequency and quantity of alcohol consumption. This approach facilitated the collection of day-by-day reports on alcohol and drug use throughout the entire study period. The data were used to derive PDA and a secondary outcome, the percentage of days of heavy drinking (PHDD), with a heavy drinking day defined as any day consuming ≥5 standard drinks for men or ≥4 drinks for women. Breathalyzer screens and urine samples were obtained at in-person visits (breathalyzers were only for participants enrolled prior to COVID-19 pandemic restrictions on exhaled breath samples), to provide biological indicators of recent alcohol and/or drug use.

#### 2.4.4 Short inventory of problems-revised Spanish (SIP-RS)

The SIP-RS is the Spanish version of the 15-item SIP, a self-report instrument that assessed the frequency of consequences due to alcohol use, instructing participants to rate how often each of the listed consequences happened to them over the past two months (NB: the timeframe was modified from the original SIP-RS measuring past three months). Items are rated from 0 (“never”) to 3 (“daily or almost daily”), with item responses summed to produce a total score ranging from 0 to 45. Internal consistency of the SIP-RS in this sample was excellent at each timepoint administered (Cronbach's *α* > 0.95 at all timepoints) (49).

#### 2.4.5 Coping strategies scale (CSS)

An abbreviated 17-item version of the original CSS that has been validated in prior studies (54) was translated into Spanish and used to measure participants' self-reported frequency of several cognitive and behavioral strategies for avoiding alcohol use over the past week. Each item is rated from 0 (“never”) to 4 (“all the time”), with item responses summed to produce a total score reflecting coping strategy usage. Internal consistency of the Spanish version of the CSS in this sample was excellent at all timepoints (Cronbach's *α* > 0.90 at each timepoint) (55, 56).

#### 2.4.6 Treatment satisfaction

Participants completed a brief survey at the end of the 8-week treatment period assessing satisfaction with treatment received, and those assigned to CBT4CBT-SA completed an additional 13-item satisfaction survey (30) evaluating various aspects of the program, with item response scales varying from Likert-type scales ranging from 1 (indicating low satisfaction/agreement) to 5 (indicating high satisfaction/agreement) or binary (yes/no) or ranked ordered responses.

### 2.5 Data analyses

All analyses were performed using SPSS, version 29.01.0 (IBM, Inc.). Statistical significance for two-sided tests was set at *p* < 0.05*.* Demographic and baseline descriptive variables, as well as treatment adherence, were evaluated across treatment condition with chi-square or analysis of variance (ANOVA). For those assigned to CBT4CBT-SA, we examined usability data including the mean number of modules completed, the average amount of time (in minutes) to complete each module, the percentage of those who completed all 7 modules, and the mean number of homework exercises completed.

The principal strategy for assessing the efficacy of the study treatments on the primary outcome (PDA) over time was longitudinal random effects regression using the maximum likelihood approach for handling missing data, with time modeled by week during the 8-week treatment period and monthly during the 6-month follow-up. This approach was also used for the secondary drinking outcome, PHDD. The percentage of subjects with no heavy drinking days [PSNHDD; (57)] in the final month of treatment was evaluated as a secondary endpoint indicator of treatment efficacy using chi-square analysis. Other secondary outcomes included change in SIP-RS total scores and CSS total scores. All analyses were conducted for the full intention-to-treat (ITT) sample, using all available data (*n* = 51). Separate analyses were conducted for the subsample of participants who were deemed *treatment completers* (defined *a priori* as those attending at least 5 sessions/modules within 8 weeks).

## 3 Results

### 3.1 Participants

Table 1 presents demographic and baseline characteristics by treatment condition. The sample was 31% female with a mean age of 42 years. Less than half (47%) completed high school, 37% were unemployed, and 33% reported being referred to treatment by the criminal justice system. There was a difference by treatment condition in the percentage of participants who were never married/living alone, with 83% assigned to ST + CBT4CBT-SA compared to 54% assigned to ST (*Χ*^2^ = 4.79, *p* = .03). Only 4% of the sample were born in the continental United States; the largest percentage (39%) reported being born in Mexico, followed by 27% born in Puerto Rico, 22% in a Central American country, 6% in South America, and 1 participant reported being born in a Caribbean Island. Approximately 41% met DSM-5 criteria for current major depressive disorder, 18% met criteria for current PTSD, and 13% for current anxiety disorder.

**Table 1:** Demographic and baseline characteristics across treatment conditions.

Treatment condition	ST *n* = 28	ST + CBT4CBT-SA *n* = 23	Total *n* = 51	
*Categorical variables*	*n* (%)	*n* (%)	*n* (%)	*Χ^2^*
Female	11 (39.3)	5 (21.7)	16 (31.4)	1.81
Client Origin of birth^a^				4.17
Puerto Rico	7 (25.0)	7 (30.4)	14 (27.4)	
United States	1 (3.6)	1 (4.3)	2 (3.9)	
South American country	1 (3.6)	2 (8.7)	3 (5.9)	
Mexico	14 (50.0)	6 (26.1)	20 (39.2)	
Other Central American country	5 (17.9)	6 (26.1)	11 (21.6)	
Caribbean Island	0 (0)	1 (4.3)	1 (2.0)	
Completed High School	12 (42.9)	12 (52.2)	24 (47.1)	0.44
Never married/living alone	15 (53.6)	19 (82.6)	34 (66.7)	4.79*
Unemployed	10 (35.7)	9 (39.1)	19 (37.3)	0.06
Referred by criminal justice syst	7 (25.0)	10 (43.5)	17 (33.3)	1.94
Psychiatric condition from SCID-5 (current)				
PTSD	3 (11.5)	6 (26.1)	9 (18.4)	1.72
Anxiety Disorder	4 914.3)	3 (13.0)	7 (13.7)	0.02
Major Depressive Disorder	10 (35.7)	11 (47.8)	21 (41.2)	0.77
AUD Severity Rating				
Mild	0 (0)	1 (4.3)	1 (2.0)	
Moderate	7 (25.0)	6 (26.1)	13 (25.5)	
Severe	21 (75.0)	16 (69.6)	37 (72.5)	1.28
*Continuous variables*	*M (sd)*	*M (sd)*	*M (sd)*	*F*
Age	39.5 (13.0)	44.8 (11.9)	41.9 (12.7)	2.26
Days of alcohol use past 28	9.1 (9.3)	7.7 (7.3)	8.5 (8.4)	0.33
Days of heavy drinking past 28	7.0 (8.7)	5.4 (6.4)	6.3 (7.7)	0.57
Drinks per drinking day past 28	8.6 (5.9)	10.9 (14.1)	9.6 (10.4)	0.62
AUDIT Score	20.3 (9.2)	18.9 (7.7)	19.7 (8.5)	0.34

* *p* *<* *.05.*

^a^ Race not reported because 90% of the sample self-identified as Hispanic only.

ST, standard treatment; CBT4CBT-SA, computer-based training for cognitive behavioral therapy—Spanish alcohol version; SCID, structured clinical interview for DSM-5; PTSD, post-traumatic stress disorder; AUD, alcohol use disorder; AUDIT, alcohol use disorder identification test.

There were no differences across treatment conditions in terms of alcohol use at baseline. Most of the sample (73%) met DSM-5 criteria for severe AUD; mean (SD) PDA during the 28-day period prior to study enrollment was 69.7% (30.0) and PHDD was 22.5% (27.6), and participants reported 9.6 (10.4) drinks per drinking day. The mean (SD) score on the AUDIT at baseline was 19.7 (8.5), indicating likelihood of moderate to severe AUD.

### 3.2 Treatment engagement, retention, and safety

Participant flow is presented in Figure 1 using a CONSORT flow diagram. A total of 64 individuals provided written informed consent and were screened for eligibility. Of these, 51 were deemed eligible, completed baseline assessments, and were randomized to a treatment condition (ST = 28; ST + CBT4CBT-SA = 23). When the COVID-19 pandemic shutdowns started in the U.S. (March 2020), 7 participants had already finished the study treatment period; 4 were active when all treatment and research visits transitioned to telephone or video-based teleconference; and 40 were recruited and enrolled after March 2020.

Treatment engagement and retention were excellent across conditions (Table 2). All randomized participants (*n* = 51) attended at least 1 treatment session, with 63% (*n* = 32) of the sample deemed treatment completers (ST = 57%; ST + CBT4CBT = 70%). Participants completed an average of 52 out of 56 days in the protocol; 84% remained active in treatment at the end of the 8-week treatment period. There were no differences across conditions in terms of the number of ST sessions completed. On average, participants completed 5.2 ST sessions, with mean (SD) of 3.1 (2.6) individual sessions and 2.0 (3.0) group sessions during the 8-week period. Those assigned to ST + CBT4CBT-SA completed a mean (SD) of 5.3 (2.1) CBT4CBT-SA modules, with 52.2% completing all 7 modules in the program. On average, participants spent approximately 48.4 (SD = 14.3) minutes completing each module. Rates of engagement and completion of CBT4CBT-SA modules are comparable to prior studies of CBT4CBT.

**Table 2:** Treatment Exposure, serious adverse events, and outcomes by treatment condition.

	ST *n* = 28	ST + CBT4CBT-SA *n* = 23	Total *n* = 51	
*Treatment Exposure*	n (%) or *M (sd)*	n (%) or *M (sd)*	n (%) or *M (sd)*	*Χ^2^* or *F*
Initiated treatment (yes)	28 (100)	23 (100)	51 (100)	0
Days in treatment (out of 56)	52.5 (11.0)	51.7 (10.8)	52.2 (10.8)	0.61
Completed treatment protocol (yes)	16 (57.1)	16 (69.6)	32 (62.7)	0.83
Active in treatment at week 8 (yes)	24 (85.7)	19 (82.6)	43 (84.3)	0.09
Number of sessions attended	4.8 (2.2)	5.6 (3.8)	5.2 (3.0)	0.95
Individual counseling sessions	3.3 (2.6)	2.9 (2.8)	3.1 (2.6)	0.30
Group counseling sessions	1.5 (2.3)	2.7 (3.5)	2.0 (3.0)	2.25
Number of CBT4CBT-SA modules completed	–	5.3 (2.1)	–	–
Completed all 7 CBT4CBT-SA modules (yes)		12 (52.2)		
*Serious Adverse Events (SAE)*	*n* (%) or	*n* (%) or	*n* (%) or	*Χ^2^*
Participants with ≥1 SAE, psychiatric or substance use related	2 (7.1)	1 (4.3)	3 (5.9)	
Participants with ≥1 SAE, medical issues	0	3 (13.0)	3 (5.9)	
*Secondary Alcohol Use Outcomes*	n (%) or *M (sd)*	n (%) or *M (sd)*	n (%) or *M (sd)*	*Χ^2^* or *F*
Percentage of days abstinent (56 days)	76.5 (25.4)	88.7 (12.3)	81.9 (21.4)	4.31*
Percentage of heavy drinking days (56 days)	15.2 (22.7)	6.4 (10.3)	11.4 (18.7)	2.86
Percentage of subjects with no heavy drinking days in final month	13 (46.4)	11 (50)	24 (48)	0.06
Percentage of urine positive (EtG)^a^	36.5 (44.1)	33.1 (33.6)	35.2 (39.7)	0.04

* *p* *<* *.05.*

^a^ Urine collected on a subsample of participants (*n* = 26 total).

ST, standard treatment; CBT4CBT-SA, computer-based training for cognitive behavioral therapy—Spanish alcohol version; EtG, ethyl glucuronide.

There were six serious adverse events that warranted hospitalization reported during the trial; two were reported for those assigned to ST (7.1%), with four reported for those assigned to ST + CBT4CBT-SA (17.4%). These included hospitalization for alcohol-related symptoms/detoxification, mental health (suicidal ideation), or medical issues (e.g., infection, kidney stones). None of these were determined to be related to study treatments. There was one participant death during the trial, who was assigned to ST + CBT4CBT-SA. Cause of death was unknown; this event was unexpected and deemed unrelated to study treatment.

### 3.3 Treatment effect on alcohol use outcomes during treatment period

**Figure 2:**
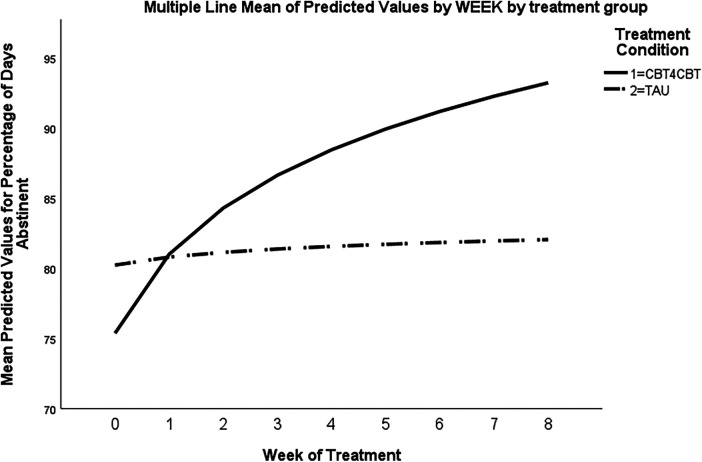
Multiple line mean of predicted values by WEEK by treatment group.

See Supplementary Table 1 for the mean PDA at each time point across treatment conditions. For the ITT sample (*n* = 51), results of random effects regression analyses for the primary outcome (PDA by week) during the 8-week treatment period did not indicate a significant change in PDA over time (effect for time, *t*_405.01_ = 1.47, *p* = .14), nor significant change over time by treatment condition (treatment by week, *t*_406.07_ = 1.05, *p* = .29). However, for the subsample of treatment completers (*n* = 32), results indicated a significant change over time in PDA by treatment condition (treatment by week, *t*_258_ = 2.56, *p* = .01), with those assigned to ST + CBT4CBT-SA showing a greater increase in PDA by week compared to ST (Figure 2). With respect to heavy drinking in the ITT sample, results for PHDD by week during the 8-week treatment did not indicate significant change over time (effect for time, *t*_405.01_ = 0.44, *p* = .66), nor change by treatment condition (treatment by week, *t*_406.23_ = −0.91, *p* = .36). Results were non-significant for PHDD in the subsample of treatment completers.

Results of ANOVA evaluating PDA over the entire treatment period (56 days) indicated those assigned to ST + CBT4CBT-SA reported a greater PDA compared to ST (88.7% vs. 76.5%; *F*_1,48_ = 4.31, *p* = .04). Those assigned to ST + CBT4CBT-SA also reported lower PHDD compared to ST, although this difference was non-significant (6.4% vs. 15.2%; *F*_1,48_ = 2.86, *p* = .10). In the final month of the treatment period, the PSNHDD did not differ across treatment conditions (ST: *M* *=* 46.4% vs. ST + CBT4CBT-SA *M* = 50%; *Χ^2^* = 0.06, *p* = .80). There was no difference in the percentage of urine samples that were positive for Ethyl glucuronide (EtG) across treatment conditions during the study period (ST: *M* *=* 36.5% vs. ST + CBT4CBT-SA *M* = 33.1%; *Χ^2^* = 0.04, *p* = .84), although urine samples were only collected on a subsample of participants (*n* = 26) due to restrictions on in-person visits during COVID-19 pandemic.

### 3.4 Treatment effect on alcohol use outcomes during follow-up period

During the 6-month follow-up period, representing study months 3–8, results of random effects regression in the ITT sample did not indicate a significant change in PDA by month (effect for time, *t*_221.50_ = −0.94, *p* = .35), nor significant change over time by treatment condition (treatment by week, *t*_221.74_ = 1.48, *p* = .14). This was also true in the subsample of treatment completers. Change in PHDD during the 6-month follow-up period was also non-significant.

However, when change in drinking outcomes were examined by month across the full study period, representing months 0–8, results of random effects regression (with month log transformed) in the ITT sample indicated a non-significant but greater increase in PDA over time for those assigned to ST + CBT4CBT-SA compared to ST (treatment by month, *t*_379.39_ = 1.73, *p* = .08). In the subsample of treatment completers, the greater increase in PDA for those in ST + CBT4CBT-SA compared to ST was statistically significant (treatment by month, *t*_239.85_ = 2.50, *p* = .01). Results also showed a statistically significantly greater decrease in PHDD by month over the 8-month study period for those assigned to ST + CBT4CBT-SA compared to ST in the ITT sample (treatment by month, *t*_379.10_ = −2.11, *p* = .04), and non-significantly greater decrease in the subsample of treatment completers (treatment by month, *t*_244.72_ = −1.89, *p* = .06).

### 3.5 Treatment effect on adverse consequences and coping skills

Results of repeated measures ANOVA indicated a significant reduction in SIP-RS scores from baseline to end-of-treatment (week 8) for the sample overall (*t* = 3.77, *p* < .01), but no differential reduction by treatment condition. Mean (SD) scores reduced from 16.1 (10.9) at baseline to 11.8 (12.1) at end-of-treatment, indicating an overall reduction in self-reported adverse consequences from alcohol. Results of random effects regression that included all timepoints up to 6-month follow-up did not indicate a significant effect of time or time x treatment condition. This was also true for the subsample of treatment completers.

In terms of change in participants' reported use of coping skills for avoiding alcohol, results of analyses that included all timepoints from baseline to 6-month follow-up did not indicate a significant effect of time or time x treatment condition, either in the ITT sample nor the subsample of treatment completers.

### 3.6 Treatment satisfaction

Only a subsample of participants (*N* = 37) completed the brief satisfaction survey at the end of the treatment period, due to modifications to reduce the length of the assessment battery during the COVID-19 pandemic. Of those completing the survey, overall ratings of satisfaction with treatment were high and did not significantly differ across the treatment conditions. In response to the question “how satisfied are you with the treatment you received?”, 56% of those assigned to ST + CBT4CBT-SA reported “very satisfied” compared to 61% assigned to ST (*Χ^2^* = 1.50, *p* = .68). Also, 56% of those assigned to ST + CBT4CBT-SA responded “I'm much better” compared to 29% assigned to ST in response to the question “*how would you describe the changes that occurred in your life since you began participating in this study?*” (*Χ^2^* = 5.56, *p* = .13). In terms of satisfaction with the CBT4CBT-SA program, mean ratings were at least “4—moderately satisfied” out of the 5-point scale for nearly all items. For instance, 94% responded at least “4—moderately satisfied” in response to the question “how satisfied are you with the overall content of the computer program?” Also, 94% responded they “agree” or “totally agree” with the statements, “*it was easy to understand the narrator of the program*”, and “*the material presented through the computer applies to my life*.” All participants (100%) reported they “agree” or “totally agree” with statements that the computer program was a valuable learning tool, that there were enough interactive components, and that the computer program helped me think about my alcohol use in a different way.

## 4 Discussion

In this trial, a newly developed, culturally adapted, web-based version of CBT targeting alcohol use for Spanish speakers appeared to be safe, feasible, and had preliminary benefits at improving alcohol use outcomes when added to standard outpatient treatment. Among a sample of 51 Spanish-speaking adults with AUD enrolled in treatment at a community-based outpatient facility, those who completed the CBT4CBT-SA program reported more days of abstinence from alcohol over an 8-week period than those completing standard care alone. Although the primary *a priori* hypothesis demonstrating a greater increase in PDA by week during the treatment period for those assigned to CBT4CBT-SA was not supported in the full ITT sample, it was evident in the sample of those who completed at least five sessions, per-protocol. Also, when alcohol use outcomes were examined for the entire 8-month study period, those assigned to CBT4CBT-SA showed a greater increase in days abstinent per month and a greater reduction in heavy drinking days per month than those provided with standard care only. Overall, these findings are consistent with trials supporting the efficacy of the English-version of this web-based CBT program as an adjunct to standard care for alcohol and drug use. CBT4CBT-SA may be a promising approach toward making linguistic and culturally adapted evidence-based treatment for AUD more accessible for Hispanic populations.

The past decade has seen exponential growth in the development and availability of digital tools targeting a range of mental and physical health conditions, yet relatively few products have undergone rigorous clinical evaluation demonstrating safety and efficacy through clinical trials (58). This is especially evident in the area of AUD, as reviews have indicated that most available mobile apps targeting alcohol do not integrate evidence-based content (59). The CBT4CBT-SA program created and tested here included: (1) core components based on manualized CBT for AUD (60), (2) a systematic process of content development that was successful at conveying CBT skills in other CBT4CBT versions (61, 62), including in Spanish (63), (3) an RCT that included an active treatment comparison condition, (4) evaluation in a treatment-seeking clinical sample with AUD, and (5) follow-up assessment with >80% data availability. Despite a small sample size that didn't reach the target estimate to have sufficient power to detect a medium-sized effect on the primary outcome, the findings regarding a significant effect of CBT4CBT-SA on PDA for treatment completers during the 8-week treatment period and across the full 8-month study period are notable. Moreover, those assigned to CBT4CBT-SA increased PDA from 73% at baseline to 93% at month 8, representing a 27% increase in non-drinking days between those time points. Future studies are needed to replicate this effect in a larger sample.

The COVID-19 pandemic, which forced the temporary closure of our medical offices less than one year after recruitment began for this trial, negatively impacted enrollment during the period of funding yet also highlighted the benefits of a web-based intervention. When in-person treatment services were forced to transition to remote delivery via phone or videoconference, likely affecting the scope and quality of standard treatment services, the format and delivery of CBT4CBT-SA was virtually unaffected. Access to the CBT4CBT-SA program remained available 24/7 and the high-quality delivery of CBT skills was maintained regardless of the access location. Those provided with access to the program showed high rates of engagement with the program, completing on average 5 out of the 7 modules, engaging for approximately 48 min per module, with 52% completing all 7 modules. Satisfaction ratings were also consistently high among participants. This is highly important, as the field of digital mental health treatment is marked by low rates of engagement with the intervention, hindering the conclusions regarding benefits (64). Engagement with CBT4CBT-SA was supported in this study through weekly assessment visits with bilingual research staff that included a check-in regarding module completion and/or technical support. Prior work suggests that human support is effective for improving engagement rates and generating evidence for efficacy of mental health apps (65, 66).

The differential impact of treatment condition on alcohol use outcomes did not appear to extend to reductions in consequences of alcohol, nor to increases in frequency of self-reported coping skills. During the 8-week treatment period, there was an overall reduction in mean adverse consequences from alcohol reported in the sample overall, but no significant difference by treatment condition. When the 6-month follow-up period was included, there did not appear to be a significant change over time in reported adverse consequences from alcohol. This is consistent with results in the ITT sample regarding non-significant change in days of alcohol abstinence over the full 8-month study period. However, the frequency of adverse consequences did not significantly change over time in the subsample of treatment completers as was the case for days of alcohol abstinence. It may be that negative consequences of alcohol (e.g., money problems because of alcohol) do not change at the same rates as changes in days of alcohol use. Surprisingly, the frequency of coping skills also did not appear to increase over time during the study period. As the content of CBT4CBT-SA is focused on conveying generalizable CBT-based coping strategies for avoiding alcohol, one might presume that a change in alcohol use was accompanied by a change in coping skills. More work is needed to uncover potential mediators of CBT4CBT-SA's effect on alcohol use, particularly for those who completed the treatment protocol.

This study's findings are limited by the small sample size that was below target based on power estimations, which was primarily due to the impact of COVID-19 pandemic on enrollment. This also affected the power to evaluate outcomes by gender or other potential baseline moderators such as AUD severity or psychiatric disorder. Another limitation is the differential time/attention across treatment conditions, as those assigned to ST + CBT4CBT-SA received access to an additional intervention that resulted in a potentially higher treatment “dose” each week during the 8-week treatment period. A future study should examine a reduced dose of ST for those with access to CBT4CBT-SA or a comparison condition that includes web-based educational materials to balance the time/attention across conditions. Also, drinking outcomes were based entirely on self-report, as the planned collection of biological indicators of alcohol use (breathalyzer and urine EtG testing) were limited or eliminated due to the impact of COVID-19 on exhaled breath or restrictions on in-person visits. Lastly, most of this Hispanic sample were of Mexican or Puerto Rican descent, limiting the generalizability to other samples of US Hispanic individuals that are more heterogeneous. Further attention to the potential influence of birthplace and years of residence in the continental United States may serve to identify subgroup differences and advance the literature on alcohol use within this population.

To our knowledge, this is the first randomized trial to evaluate a culturally adapted, Spanish-language, digital CBT intervention for Hispanic individuals with AUD. As access to evidence-based CBT for alcohol use may be limited for Spanish-speaking populations, the CBT4CBT-SA program offers the potential to provide an inexpensive, scalable intervention that can be implemented in standard treatment programs and other non-specialty settings. Given the need for evidence-based behavioral health treatments for alcohol use among the growing population of Hispanic individuals with limited English proficiency in the US, and the lack of adequately trained bilingual providers, this digital program may be an appealing treatment option for patients and service providers alike.

## Data Availability

The raw data supporting the conclusions of this article will be made available by the authors, without undue reservation.
