# Supplementary material for: A digital cognitive behavioral therapy program culturally adapted for Spanish-speaking individuals with alcohol use disorder: a stage 1 randomized clinical trial
Source: Front Digit Health. 2026 Apr 21;8:1729049. doi: 10.3389/fdgth.2026.1729049 (PMC13139163; doi:10.3389/fdgth.2026.1729049)
Supplement: Supplementary file 1 [file Table1.docx]

|  | **ST** | | **ST + CBT4CBT-SA** | |
| --- | --- | --- | --- | --- |
| **Percentage of Days Abstinent (PDA) by week (Active Treatment)** | *M (sd)* | n | *M (sd)* | n |
| Baseline | 67.0 (34.6) | 28 | 73.3 (25.7) | 23 |
| Week 1 | 79.6 (31.0) | 28 | 88.2 (19.6) | 23 |
| Week 2 | 78.1 (29.2) | 28 | 83.2 (22.7) | 23 |
| Week 3 | 77.6 (31.7) | 28 | 91.3 (12.0) | 23 |
| Week 4 | 71.4 (30.6) | 28 | 90.3 (15.5) | 22 |
| Week 5 | 72.5 (28.3) | 28 | 92.9 (12.3) | 22 |
| Week 6 | 80.1 (29.7) | 28 | 89.6 (14.7) | 22 |
| Week 7 | 77.0 (31.7) | 28 | 87.0 (24.1) | 22 |
| Week 8 | 75.5 (31.6) | 28 | 84.4 (22.4) | 22 |
| **PDA by month**  **(Follow-up)** | *M (sd)* | n | *M (sd)* | n |
| Month 3 | 77.5 (29.3) | 27 | 89.0 (10.6) | 21 |
| Month 4 | 76.9 (29.3) | 27 | 89.9 (10.6) | 21 |
| Month 5 | 76.3 (29.3) | 27 | 90.7 (10.6) | 21 |
| Month 6 | 75.7 (29.3) | 27 | 91.5 (10.6) | 21 |
| Month 7 | 75.2 (29.3) | 27 | 92.3 (10.6) | 21 |
| Month 8 | 74.6 (29.3) | 27 | 93.1 (10.6) | 21 |

Supplement Table 1. Percentage of Days Abstinent from Alcohol (PDA) During Active Treatment and Follow-Up Across Conditions

Note: ST = Standard Treatment; CBT4CBT-SA = Computer-Based Training for Cognitive Behavioral Therapy – Spanish Alcohol version
